# An Evaluation of Emergency Detention Certificates in NHS Lanarkshire: A Quality Improvement Project

**DOI:** 10.1192/bjo.2026.11262

**Published:** 2026-06-30

**Authors:** Lewis Allan, Clare Langan, Andrew Donaldson

**Affiliations:** NHS Lanarkshire, Glasgow, United Kingdom

## Abstract

**Aims:**

An Emergency Detention Certificate (EDC) can be issued by any registered medical practitioner in Scotland. An EDC authorises detention of a person with a likely mental disorder in hospital for up to 72 hours. The granting of an EDC poses significant restrictions on a patient’s liberty and rights. It is therefore crucial that the completion of EDCs meet both sufficient clinical and legal requirements.

The aim of this quality improvement project is to systematically evaluate EDCs issued across 2 sites in NHS Lanarkshire over a defined time period to identify any areas for improvement. Ultimately, we aim to improve compliance with completion of EDCs in line with the principles of the Mental Health Act.

**Methods:**

EDCs across NHS Lanarkshire in a 6-month period were obtained. A standardised scoring system was devised to evaluate each EDC against a minimum criteria. The standardised scoring system utilised by the three reviewers generated a total score of 28, with a higher score reflecting a higher quality of EDC completion.

Data regarding the practitioner who completed the EDC and if Mental Health Officer consent was sought was also obtained.

**Results:**

A total of 125 EDCs were identified. 8 EDCs utilised the incorrect form and so were excluded. 117 EDCs were reviewed. We found a range of scores from 16 to 28. The average score was 23.

EDCs were completed by doctors from a range of settings. 51.3% of EDCs were completed by psychiatry resident doctors or psychiatry consultants, 11.1% by GPs and 37.6% by resident doctors in medical wards. The average score for EDCs completed by doctors working in psychiatry was higher at 24.2. EDCs completed by doctors working in medical wards and by GPs scored 22.1 and 20.2 respectively. 35% of EDCs completed had MHO consent. Of the EDCs that had MHO consent, 53.6% EDCs were placed by doctors working in psychiatry.

**Conclusion:**

Completion of EDCs remains suboptimal and there is still work to be done in improving the quality of EDC completion. EDCs are generally completed to a higher standard by doctors working in psychiatry which may be due to a range of factors includingteaching on EDC completion and regular informal feedback. MHO consenting to the granting of an EDC continues to remain low at 35%.

This quality improvement project highlights the need for teaching and education tools as anintervention to improve the standard of completion of EDCs.

